# The complete mitochondrial genome of *Chrysomya nigripes* (Diptera: Calliphoridae)

**DOI:** 10.1080/23802359.2019.1687028

**Published:** 2019-11-13

**Authors:** Ze-Ya Zhang, Li-Pin Ren, Jing Leng, Yan-Jie Shang, Guang-Hui Zhu

**Affiliations:** a Department of Forensic Medicine, Shantou University Medical College, Shantou, China;; b Department of Forensic Science, School of Basic Medical Sciences, Central South University, Changsha, China;; c Cardiovascular research center, Shantou University Medical College, Shantou, China

**Keywords:** Chrysomya nigripes, mitochondrial genome, species identification, phylogenetic analyses

## Abstract

*Chrysomya nigripes* (Diptera: Calliphoridae) is a blow fly species of forensic importance. Here we demonstrated the complete mitochondrial genome of this species for the first time. Phylogenetic analyses indicated that entire mitochondrial genome sequences can provide more useful information for distinguishing *C. nigripes* from the other species.

*Chrysomya nigripes* (Diptera: Calliphoridae) (Aubertin, 1932) is a forensically important blow fly species, which is widespread in Asian and Australasian (O’Flynn 1983; Sukontason et al. 2005). Considering the underlying value in forensic entomology, here we report the entire mitochondrial genome sequence of *C. nigripes* for species identification and phylogenetic analysis (GenBank accession number: KT444441).

Adult specimens of *C. nigripe*s were collected in Changsha, China (28°9′N; 112°54′E) in August, 2014. All specimens were frozen to death and stored under −80 °C subsequently. The specimens were identified according to the key of morphological characteristics (Xue and Zhao 1996). The voucher specimens were assigned a unique field code with a sole code (STU20140801) and the DNA with a sole code (STU20140802) was deposited in the Zhu’s Lab (Shantou University, Shantou, China). The complete genome was amplified in eight fragments (Nelson et al. 2012), and the DNA fragments were sequenced on both strands by Sanger dideoxy sequencing method by a commercial service provider (Transduction Bio Co. Ltd. Wuhan, China). The entire genome of *C. nigripes* was circular with the length of 15,832 bp, including 22 transfer RNA genes, 13 protein-coding genes, 2 ribosomal RNA genes and a control region.

Phylogenetic analysis was conducted on 13 entire mitochondrial gene sequences from 9 species of Calliphoridae, together with another one from Muscidae family as the outgroup species. The phylogeny of family Calliphoridae based on the entire mitochondrial gene sequences was separated into several genetic clades (Figure 1). As the out group, two Muscidae samples were clustered together and definitely divided from the Calliphoridae.

**Figure 1.**
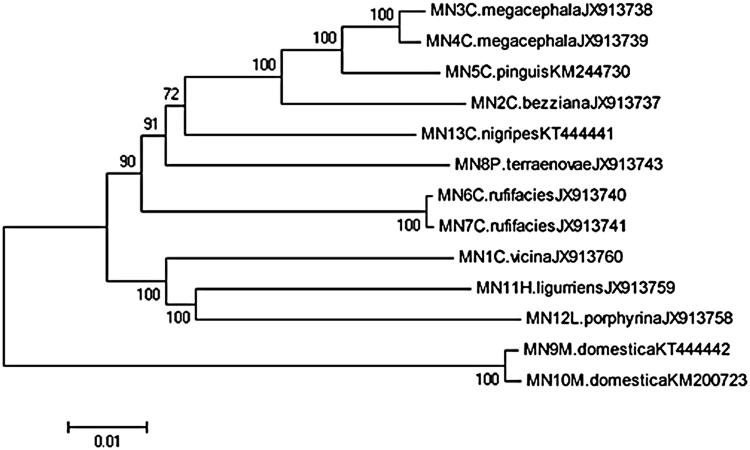
Neighbor-joining tree by maximum synthetic likelihood method based on the complete mitochondrial gene sequences from nine species of Calliphoridae.

Here we demonstrated the complete mitochondrial genome of *C. nigripes* and performed the phylogenetic analysis. The results were considered to be valuabe for the implementation of the mitochondrial database of Calliphoridae, and for establishing methods for species identification of the fly group.
